# Influences of Dietary Added Sugar Consumption on Striatal Food-Cue Reactivity and Postprandial GLP-1 Response

**DOI:** 10.3389/fpsyt.2017.00297

**Published:** 2018-01-04

**Authors:** Hilary M. Dorton, Shan Luo, John R. Monterosso, Kathleen A. Page

**Affiliations:** ^1^Neuroscience Graduate Program, University of Southern California, Los Angeles, CA, United States; ^2^Diabetes and Obesity Research Institute, University of Southern California, Los Angeles, CA, United States; ^3^Internal Medicine, Division of Endocrinology, University of Southern California, Los Angeles, CA, United States; ^4^Department of Psychology, University of Southern California, Los Angeles, CA, United States

**Keywords:** dietary sugar, brain, striatum, reward processing, GLP-1, functional magnetic resonance imaging

## Abstract

Sugar consumption in the United States exceeds recommendations from the American Heart Association. Overconsumption of sugar is linked to risk for obesity and metabolic disease. Animal studies suggest that high-sugar diets alter functions in brain regions associated with reward processing, including the dorsal and ventral striatum. Human neuroimaging studies have shown that these regions are responsive to food cues, and that the gut-derived satiety hormones, glucagon-like peptide-1 (GLP-1), and peptide YY (PYY), suppress striatal food-cue responsivity. We aimed to determine the associations between dietary added sugar intake, striatal responsivity to food cues, and postprandial GLP-1 and PYY levels. Twenty-two lean volunteers underwent a functional magnetic resonance imaging (fMRI) scan during which they viewed pictures of food and non-food items after a 12-h fast. Before scanning, participants consumed a glucose drink. A subset of 19 participants underwent an additional fMRI session in which they consumed water as a control condition. Blood was sampled for GLP-1, and PYY levels and hunger ratings were assessed before and ~75 min after drink consumption. In-person 24-h dietary recalls were collected from each participant on three to six separate occasions over a 2-month period. Average percent calories from added sugar were calculated using information from 24-h dietary recalls. A region-of-interest analysis was performed to compare the blood oxygen level-dependent (BOLD) response to food vs. non-food cues in the bilateral dorsal striatum (caudate/putamen) and ventral striatum (nucleus accumbens). The relationships between added sugar, striatal responses, and hormone changes after drink consumption were assessed using Spearman’s correlations. We observed a positive correlation between added sugar intake and BOLD response to food cues in the dorsal striatum and a similar trend in the nucleus accumbens after glucose, but not water, consumption. Added sugar intake was negatively associated with GLP-1 response to glucose. *Post hoc* analysis revealed a negative correlation between GLP-1 response to glucose and BOLD response to food cues in the dorsal striatum. Our findings suggest that habitual added sugar intake is related to increased striatal response to food cues and decreased GLP-1 release following glucose intake, which could contribute to susceptibility to overeating.

## Introduction

The average intake of added sugars in the United States has increased by 19% over the last three decades. Increases in added sugar consumption are linked to obesity, diabetes and cardiovascular disease (1–5). Excessive sugar consumption is due at least in part to the wide availability of convenient, high-sugar foods coupled with an abundance of environmental food cues that prime eating behavior (6, 7). In response to food cues, the brain recruits regions important for reward anticipation and processing, including striatal areas involved in dopaminergic signaling that motivate feeding behavior (8). Neuroimaging studies have consistently shown that striatal areas are activated in response to pictures of palatable food cues (9–11), and this response is exaggerated in obese individuals (12–14). Independent from suggested effects of obesity, chronic exposure to specific nutrients, such as sugar, may affect striatal responses to food cues. A number of studies in animal models have shown that high-sugar diets alter the striatal dopamine system (15–17), including a recent study that found that 7 months of high-sugar feeding increased basal glucose metabolism in mesolimbic reward regions independent of insulin sensitivity or weight gain in Yucatan mini pigs (18). However, it is currently unknown whether habitual dietary added sugar consumption is related to striatal food-cue reactivity in humans.

The incretin hormones glucagon-like peptide-1 (GLP-1) and peptide YY (PYY) are released in response to food intake. These hormones are known to produce anorexigenic effects through receptors concentrated in the arcuate nucleus of the hypothalamus (19, 20). Aside from effects on hypothalamic appetite circuits, GLP-1 and PYY also regulate food intake through their action on regions associated with food reward and learning (21, 22). Neuroimaging studies in humans have shown that the infusions of GLP-1 and/or PYY reduce brain responses to food cues within cortical–striatal areas involved in the regulation of eating, and these reductions in neural food-cue reactivity were associated with a decrease in ratings of appetite (23–25). These findings suggest a relationship between increases in circulating levels of incretin hormones, reductions in food-cue reactivity, and discernable feelings of satiety.

GLP-1, in particular, has emerged as a possible mediator of processing food reward and other rewarding stimuli through its action in the mesolimbic circuit (26, 27). Interestingly, studies indicate that energy-dense diets may reduce GLP-1 signaling in the brain (28, 29). Recently, Richards et al. reported that a high-fat diet resulted in decreased numbers of intestinal L-cells (the cells that secrete GLP-1) and a smaller GLP-1 response to nutrient exposure (29). However, whether chronic dietary sugar consumption affects endogenous GLP-1 secretion in humans is not yet known.

The aim of this study was to examine associations between dietary added sugar intake and (1) striatal responsivity to food cues as well as (2) the rise in circulating hormones, GLP-1 and PYY, in response to oral glucose in healthy-weight volunteers. Due to specific interest in the dorsal striatum and nucleus accumbens, we used a region-of-interest (ROI)-based analysis focusing on these regions. We used a standardized 75 g oral glucose load that has been previously shown to stimulate gut hormone secretion and to diminish food-cue reactivity (30, 31) to test the hypothesis that increases in dietary added sugar intake would be associated with greater striatal responses to palatable food cues as well as decreased systemic GLP-1 and PYY responses to glucose ingestion.

## Materials and Methods

### Participants

Twenty-two lean, young adult volunteers (12 females; 10 males) participated in the study. Participants were lean (BMI 22.6 ± 1.9 kg/m^2^), right-handed, nonsmokers, weight stable for 3 months, non-dieters, not on any medication (except oral contraceptives), with normal or corrected-to-normal vision and no history of diabetes, eating disorders, or other medical diagnoses. During the course of the study, participants were asked to adhere to their usual diet and physical activity levels. Participants provided written informed consent compliant with the University of Southern California Institutional Review Board.

### Experiment Overview

Each participant attended an initial screening visit to assess eligibility for participation in the study. During the screening visit, we collected demographic information, and anthropometric measurements including height (cm), weight (kg), waist and hip circumferences (cm), and total body fat percentage using bioelectrical impedance analysis (Model no. SC-331S, TANITA Corporation of America, Inc.). 24-h dietary intake and physical activity recalls were obtained at the screening visit. In addition, over the course of 2 months, an additional three to six dietary recalls were obtained *via* in-person interviews.

Magnetic resonance imaging (MRI) scans were performed at the Dornsife Cognitive Neuroimaging Center of University of Southern California. Participants arrived at approximately 8:00 a.m. after a 12-h overnight fast. A baseline (fasting) blood draw was performed at approximately 8:45 a.m. After performing a T1 structural scan, participants received a standardized drink containing 75 g of glucose and 0.45 g of non-sweetened cherry flavoring dissolved in 300 ml of water. The 75 g oral glucose load has been used extensively to examine changes in appetite hormones and brain regions involved in hunger, reward, and food intake (32). Although the main aim of the study was to examine the impact of sugar intake on food-cue reactivity and satiety hormone release in response to a glucose load, a subset of 19 participants (M = 9; F = 10) also underwent a second imaging session in which they consumed 300 ml of water with flavoring as a control condition. The order of the drink sessions was randomized, and the time interval between the two sessions was between 2 and 30 days. Participants were instructed to consume the drink within 2 min. After consuming the drink, participants entered the scanner and underwent a food-cue task (described below). Another blood draw was performed after the scan at, on average, 75 min after the drink was consumed. Immediately following each blood draw, participants were asked to rate their hunger from 1 to 10 on a visual analog scale. Females underwent MRI scans during the follicular phase of the menstrual cycle. These data were collected as part of a larger study aimed at determining brain responses to sugar.

### Assessments of Dietary Intake

To assess dietary intake, we used the multipass 24-h dietary recall, a validated method that probes quantities of food and drink consumed during the previous 24 h (33, 34). A trained staff member administered each dietary recall interview, which spanned between 30 and 60 min. During the dietary recall interview, participants were asked to report all food and beverage items (including meals and snacks) they consumed during the prior 24 h. Participants were also asked to provide the amount of each item she or he consumed, approximate time of consumption, a description of the preparation method, and additional details such as brand name. Dietary recalls captured dietary intake on both weekend days and weekdays to account for individual variations in dietary intake.

### Physical Activity Assessments

We also collected information on habitual physical activity levels to control for the potential confounding effects of physical activity on brain and endocrine responses (35, 36). Physical activity data were recorded during an interview with a trained staff member using a 24-h physical activity recall (PAR). Participants were asked to report what activities they did, in 30-min increments between the hours of 7:00 a.m. and 11:59 p.m. on the previous day. Using data from each participant’s PAR, we calculated daily physical activity by summing the metabolic equivalence (MET) of each activity at each interval. We used the mean daily METs for each participant to reflect the overall level of physical activity.

### Food-Cue Task

Participants completed the food-cue task in the MRI scanner. In a randomized block design, participants were presented with a total of 12 visual food cue and non-food cue blocks using Matlab (MathWorks, Inc., Natick, MA, USA) and Psychtoolbox on an Apple laptop. Four cue images per block were presented in random order, each separated by 1 s of a blank screen. Within a block, each image was presented for 4 s. Food-cue stimuli were images of high-calorie, palatable food items such as cookies and pizza. The control stimuli were images of neutral, non-food items such as buses and staircases. The set of food and non-food cue images was matched for visual appeal for use in prior published work (37–39).

### MRI Imaging Parameters

Food cue and structural MRI data were collected using 3 T Siemens MAGNETOM Tim/Trio scanner (*N* = 12) and MAGNETOM Prisma fit MRI scanner (*N* = 10) due to a scanner upgrade in the middle of our study. Participants laid supine on the scanner bed, viewing stimuli through a mirror mounted over the head coil. Functional blood oxygen level-dependent (BOLD) signals were acquired with a single-shot gradient echo planar imaging sequence. Thirty-two 4-mm thick slices covering the whole brain were acquired using the following parameters: repetition time (TR) = 2,000 ms, echo time (TE) = 25 ms, bandwidth = 2,520 Hz/pixel, flip angle = 85°, field of view (FOV) = 220 mm × 220 mm, matrix = 64 × 64. A high-resolution 3D magnetization prepared rapid gradient echo sequence (TR = 2,530 ms; TE = 2.62 ms; bandwidth = 240 Hz/pixel; flip angle = 9°; slice thickness = 1 mm; FOV = 256 mm × 256 mm; matrix = 256 × 256) was used to acquire structural images for multi-subject registration.

### Functional Magnetic Resonance Imaging (fMRI) Data Analysis

**Figure 1:**
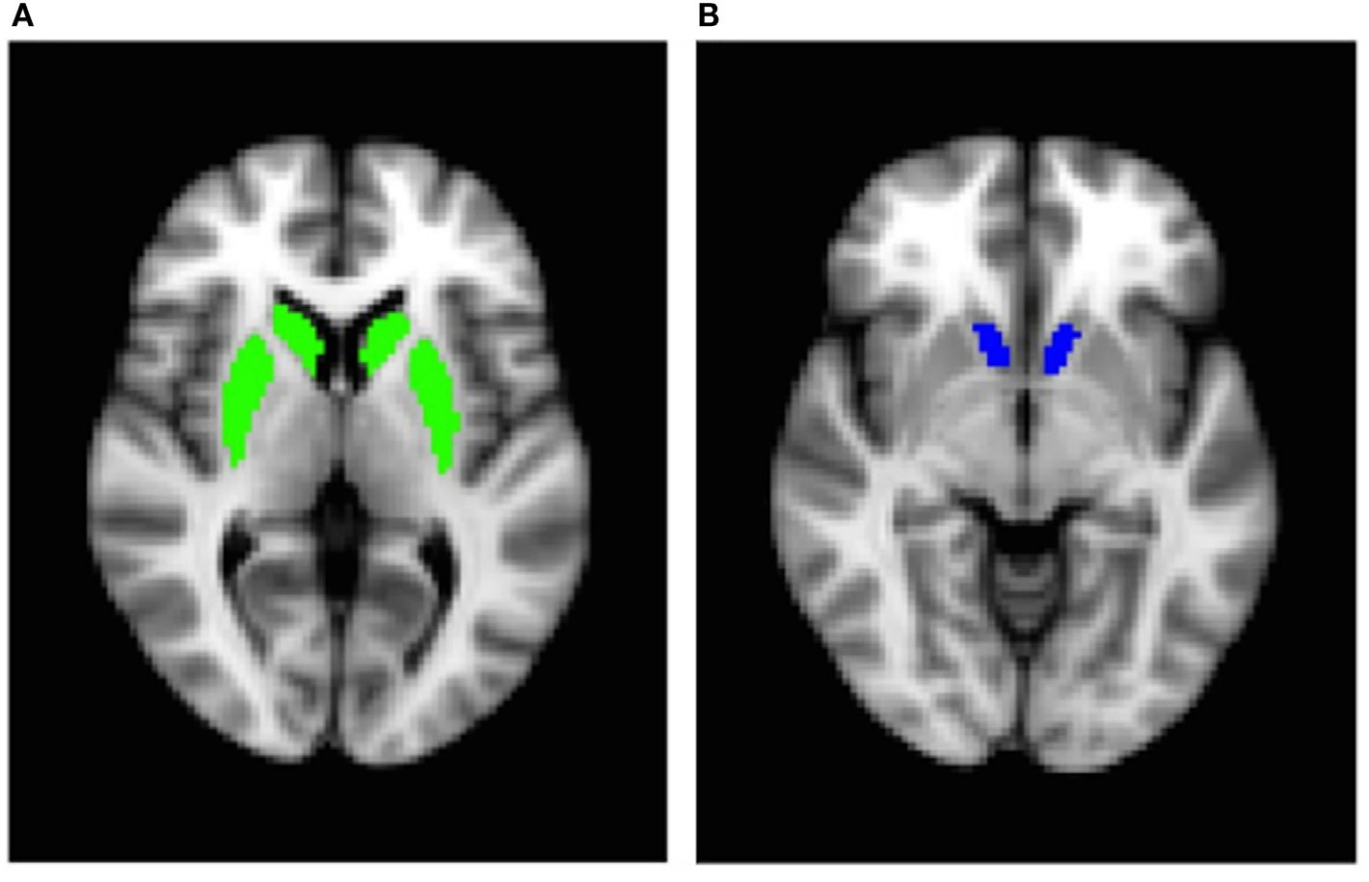
Bilateral region-of-interest masks: **(A)** dorsal striatum (caudate/putamen) and **(B)** nucleus accumbens.

To analyze fMRI data, we used several tools from the Oxford University Centre for Functional MRI of the Brain Software Library (FMRIB) (40–42). fMRI data were processed using the fMRI Expert Analysis Tool (FEAT) version 6.00. Four functional volumes (4 TRs) acquired at the beginning of each MRI session were discarded to account for magnetic saturation effects. fMRI files were preprocessed using motion correction, high-pass filtering (100 s), and spatial smoothing with a Gaussian kernel of full width at half-maximum = 5 mm. Functional data were first mapped to each participant’s anatomical image and then registered into standard space [Montreal Neurological Institute (MNI)] using affine transformation with FMRIB’s Linear Image Registration Tool to the avg152 T1 MNI template. Food and non-food events were added to the general linear model after convolution with a canonical hemodynamic response function. Temporal derivatives and temporal filtering were added to increase statistical sensitivity. For each participant, food cues vs. non-food cues contrast maps were created on the first-level analysis. An additional explanatory variable was added in the group-level analysis to account for variability due to the upgrade. Because of evidence suggesting that the striatum may be particularly affected by dietary sugar (16), we used an ROI-based approach. Anatomical, bilateral ROIs of the dorsal striatum (caudate/putamen) and the nucleus accumbens were created using the Harvard-Oxford subcortical atlas, which provides probabilistic mapping of 21 subcortical brain structures (Figures 1A,B). Percent signal change was extracted from each ROI for food vs. non-food contrast for each participant using FSL’s FEATquery, a tool within FEAT that allows the mean signal to be extracted from a given ROI mask.

### Dietary Data Analysis

Data from dietary recalls were manually checked for quality. To determine outliers, we performed linear regression analysis, using bodyweight to predict caloric intake. Residuals were standardized and examined for any values that were >3 SDs from the mean. Using this method, 102 dietary recalls were included in this analysis (an average of 4.7 recalls per participant), and none were excluded. Dietary intake data were collected and analyzed using Nutrition Data System for Research software version 2015, developed by the Nutrition Coordinating Center, University of Minnesota, Minneapolis, MN, USA (43). Using the output from this software, each participant’s dietary recall was probed for intake of overall calories, macronutrients, total sugar, and added sugar. We chose to use percent calories from added sugar as our measure of sugar intake to account for total energy intake. We calculated percent calories from added sugar by available carbohydrate from each participant’s dietary recalls and used the participant’s mean values across all recalls to represent average dietary added sugar consumption.

### Hormone Analysis

GLP-1_(7–36)_ (active) and PYY (total) were measured using Luminex multiplex technology (EMD Millipore, St. Charles, MO, USA). Change from baseline GLP-1 (pg/ml) and PYY (pg/ml) was calculated as a difference between hormone levels measured at ~75 min post drink ingestion and levels measures at the fasting blood draw.

### Statistical Analysis

All analyses were performed using R Statistical Software Version 3.1.2 (http://www.R-project.org/). Descriptive statistics were derived using the “psych” package. Spearman’s correlations were performed between average percent calories from added sugar, percent signal change in both ROIs, and GLP-1 and PYY change from baseline using the “ppcor” package. We performed Spearman’s correlations due to small sample size, which is highly sensitive to outliers. Results with *p* < 0.05 were considered significant, and imaging data were corrected for multiple comparisons (i.e., 2 regions of interest). All data are reported in mean ± SD.

## Results

### Participants

Mean age, body mass index, and body fat percentage for 22 participants are described in Table 1. Males and females did not differ in age (M = 21.5 ± 1.8; F = 21 ± 2.4; *p* = 0.5) or body mass index (M = 23.1 ± 1.5; F = 22.1 ± 2.1; *p* = 0.2), but females had higher body fat percentage than males (M = 15.9 ± 3.1; F = 24.4 ± 5.6; *p* < 0.001). Physical activity levels did not differ between males and females (M = 61.9 ± 8.6 METs; F = 61.44 ± 3.8 METs; *p* = 0.87).

**Table 1:** Participant characteristics (*n* = 22).

Characteristic	Mean ± SD
Sex	Male: *n* = 10; female: *n* = 12
Age (years)	21.2 ± 2.1
BMI (kg/m^2^)	22.6 ± 1.9
Total body fat (%)	20.6 ± 6.3

### Dietary Intake

Participants consumed an average of 1,719.1 ± 470.4 kcal/day (Table 2). Males consumed significantly more total calories per day than females (M = 1,967.8 ± 429.4; F = 1,511.78 ± 410.4 kcal/day; *p* = 0.02). However, average calories consumed from added sugar did not differ between males and females (M = 252.2 ± 127.7; F = 164.2 ± 120.8 kcal/day; *p* = 0.12), and neither did percent calories consumed from added sugar (M = 12.6 ± 6.4; F = 10.8 ± 6.7%; *p* = 0.5). In general, the percent calories from carbohydrate, fat, and protein consumed by our participants resemble the national averages for individuals in this demographic range (44).

**Table 2:** Results from 24 h dietary recalls: average energy, fat, carbohydrate, protein, total sugar, and added sugar intake (*n* = 22).

Nutrient	Unit	Mean ± SD
Energy	kcal/day	1,719.1 ± 470.4
Fat	g/day	67.4 ± 24.6
	kcal/day	606.9 ± 221.5
	% kcal	34.8 ± 7
Carbohydrate	g/day	206.7 ± 71.8
	kcal/day	826.6 ± 287.1
	% kcal	48.1 ± 10.3
Protein	g/day	72.1 ± 20.8
	kcal/day	288.5 ± 83.3
	% calories	17.2 ± 4
Total sugar	g/day	79.2 ± 34.7
	kcal/day	316.6 ± 138.8
	% calories	18.6 ± 6.9
Added sugar	g/day	51.1 ± 32.3
	kcal/day	204.2 ± 129
	% calories	11.6 ± 6.5

### Added Sugar Intake and Post-Glucose Brain Response to Food Cues

**Figure 2:**
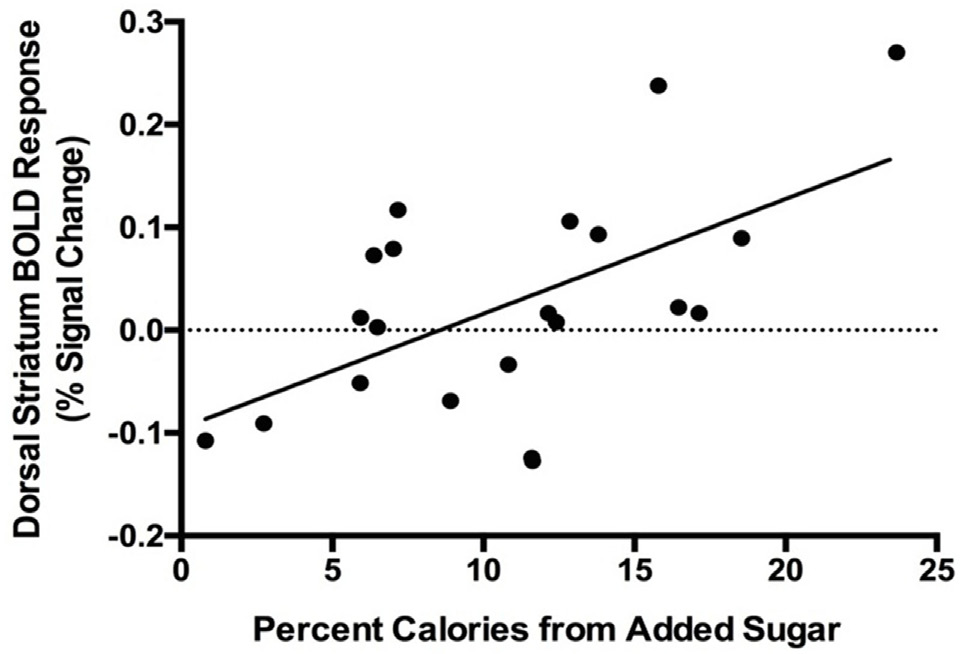
Positive correlation between blood oxygen level-dependent (BOLD) response to food cues in the dorsal striatum and percent calories consumed from added sugar (*r*_s_ = 0.55, *p* = 0.02). One male participant was excluded from analysis due to an imaging acquisition error.

Region-of-interest analysis revealed a positive correlation between percent calories consumed from added sugar and dorsal striatum response to food vs. non-food cues (*r*_s_ = 0.55, *p* = 0.02, Figure 2), and the relationship remained significant after controlling for sex, percent body fat, and average daily physical activity levels (*r*_s_ = 0.62, *p* = 0.001). We observed a trend toward a positive correlation between percent calories from added sugar and nucleus accumbens response to food vs. non-food cues (*r*_s_ = 0.41, *p* = 0.07), and controlling for sex, percent body fat, and average daily physical activity levels strengthened this relationship (*r*_s_ = 0.47, *p* = 0.03). These findings suggest that, independent of sex, adiposity, or physical activity levels, dietary added sugar consumption is associated with striatal reactivity to food cues. We did not observe a significant correlation between added sugar intake and response to food cues in the dorsal striatum (*r*_s_ = −0.22, *p* = 0.37) or nucleus accumbens (*r*_s_ = 0.29, *p* = 0.21) after ingestion of water.

### GLP-1 and PYY Responses to Oral Glucose

**Figure 3:**
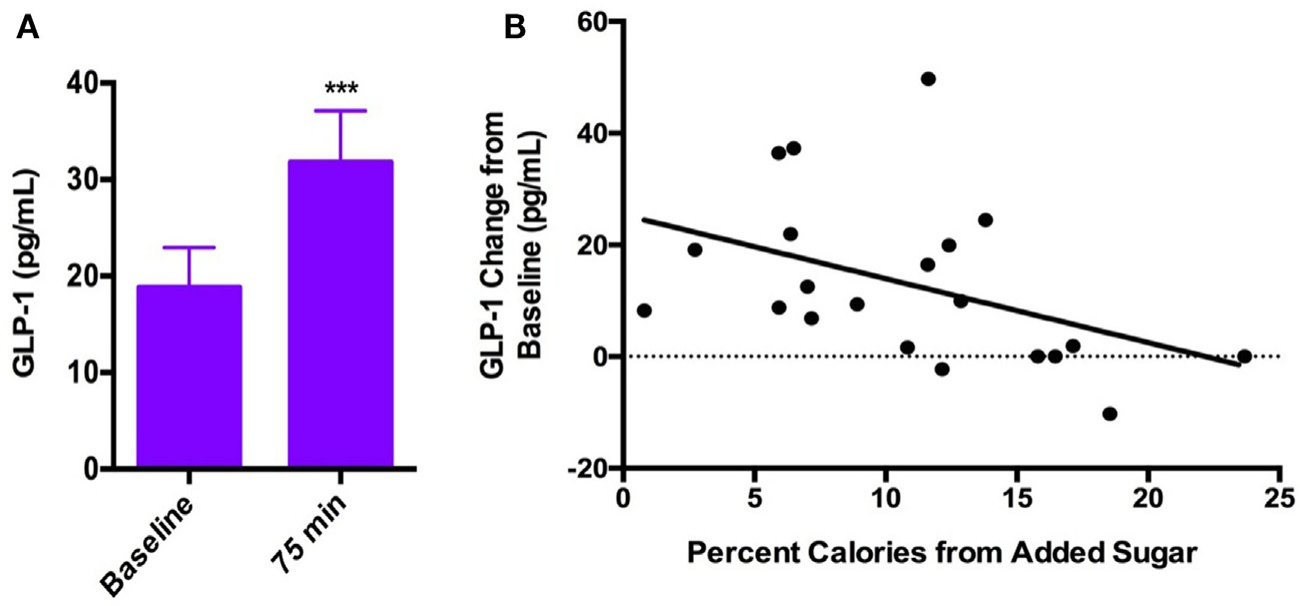
**(A)** Systemic GLP-1 increased significantly after consumption of oral glucose (Baseline = 18.8 ± 18.9 pg/ml; 75 min = 31.8 ± 24.5 pg/ml; *p* < 0.001). **(B)** Negative correlation between GLP-1 response to oral glucose and percent calories consumed from added sugar (*r*_s_ = −0.50, *p* = 0.04).

Circulating GLP-1 levels were significantly greater ~75 min after glucose consumption than at baseline (Baseline = 18.8 ± 18.9 pg/ml; 75 min = 31.8 ± 24.5 pg/ml; *p* < 0.001; Figure 3A). We observed a moderate rise in PYY levels ~75 min after glucose ingestion compared with baseline (Baseline = 78 ± 25.6 pg/ml; 75 min = 89 ± 36 pg/ml; *p* = 0.07). During the water session, there was not a significant increase in GLP-1 at the 75-min time point relative to baseline (Baseline = 18.7 ± 18.4 pg/ml; 75 min = 18.6 ± 19.3 pg/ml, *p* = 0.78). In addition, we observed a non-significant trend toward a decrease in PYY levels at 75 min relative to baseline after water (Baseline = 79.3 ± 44.7 pg/ml; 75 min = 71.7 ± 38.2 pg/ml, *p* = 0.07), likely attributable to prolonged fasted state. Hunger ratings were significantly higher 75 min after the water drink compared with the glucose drink (*p* < 0.001).

### Added Sugar Intake and GLP-1 and PYY Responses to Oral Glucose

Dietary added sugar intake was negatively correlated with the GLP-1 response to glucose ingestion (*r*_s_ = −0.50, *p* = 0.04; Figure 3B). There was no significant correlation between PYY response to glucose ingestion and dietary added sugar intake (*r*_s_ = −0.09, *p* = 0.69). In addition, we found no correlations between added sugar intake and GLP-1 or PYY response to water consumption (GLP-1: *r*_s_ = 0.12, *p* = 0.6; PYY: *r*_s_ = 0.13, *p* = 0.6).

### GLP-1 and PYY Responses to Oral Glucose and Striatal Food-Cue Reactivity

**Figure 4:**
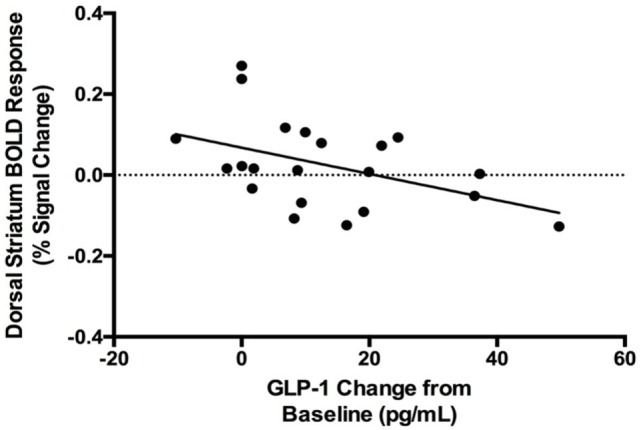
Negative correlation between blood oxygen level dependent (BOLD) response to food cues in the dorsal striatum and systemic GLP-1 response to oral glucose (*r*_s_ = −0.46, *p* = 0.04).

A *post hoc* analysis revealed that GLP-1 response to glucose was negatively correlated with the dorsal striatal response to food cues (*r*_s_ = −0.46, *p* = 0.04; Figure 4). There was no significant correlation between GLP-1 response to glucose and nucleus accumbens food-cue reactivity (*r*_s_ = −0.16, *p* = 0.5). PYY response to glucose was not correlated with either dorsal striatal or nucleus accumbens response to food cues. Neither GLP-1 response to glucose nor dorsal striatum response to food cues correlated with change in hunger ratings after glucose (GLP-1: *r*_s_ = 0.09, *p* = 0.7; dorsal striatum: *r*_s_ = −0.37, *p* = 0.1).

## Discussion

We found that increased dietary added sugar intake, independent of sex, adiposity, and physical activity, correlates with increased striatal reactivity to food cues following glucose consumption. A number of neuroimaging studies have shown that obesity is associated with increased striatal food-cue reactivity (12–14). Our findings add to the literature by demonstrating that among healthy-weight young adults, and after controlling for sex, physical activity, total energy intake, and adiposity, those who consumed greater habitual dietary added sugar had greater striatal responses to food cues in a postprandial state. It is possible that habitual consumption of added sugars could sustain the salience of external food cues and thus, the reward value of food.

Both the dorsal striatum and nucleus accumbens are associated with reward anticipation, but the dorsal striatum is often specifically associated with motivation to engage in rewarding behavior and anticipation of food and drugs (45–47). Studies in humans and animals indicate that there are hunger state-dependent changes in striatal response to both food cues and food receipt (48, 49). Participants in our study reported higher hunger ratings after consumption of water than after glucose. We found that higher added sugar intake was associated with reduced GLP-1 response to glucose. GLP-1 receptors are widely expressed in subcortical structures of the brain that relate to both homeostatic and hedonic food intake including the hypothalamus, hippocampus, amygdala, ventral tegmental area, and the nucleus of the solitary tract (50). Notably, GLP-1 receptors are expressed in the substantia nigra pars compacta, which targets the dorsal striatum through dopaminergic signaling and regulates feeding motivation (51–53).

Recently, the gut–brain axis has received attention as a crucial regulator of satiety, metabolism, and food reward (32). The vagus nerve serves as the direct connection between the gut and the brain (54). Vagal afferent neurons express GLP-1 receptors and are thought to deliver short-term satiety signals to the brain, regulating food intake, though possibly not long-term regulation of body weight (55). Animal studies suggest that impaired communication between the gut and brain leads to interrupted satiety signaling. In vagotomized rodents, peripheral administration of GLP-1 fails to reduce food intake (56). Reduced expression of GLP-1 receptor in vagal afferents leads to increases in food intake and post-feeding blood glucose levels in rats (57). Beyond homeostatic mechanisms, evidence suggests that GLP-1 may promote satiety by modulating the rewarding properties of food (58). In rats, administration of Exendin-4, a GLP-1 analog, reduced sucrose intake, diminished conditioned place preference for sweet reward, and reduced the motivation for feeding behavior (26, 59), effects that are mediated by the mesolimbic dopamine system. In humans, GLP-1 receptor blockade was shown to reduce deactivation to food cues following a meal (60). Thus, if satiety signals are impaired after consuming calories, the salience of food cues may remain high and lead to overeating and susceptibility for weight gain. A recent study reported that the dorsal striatum processes the caloric value of sugar (61). In the context of the results of our study, this process may be interrupted by reduced GLP-1 mediated satiety signaling. *Post hoc* analysis revealed that, while systemic PYY levels were not significantly correlated with striatal food-cue reactivity, participants with smaller postprandial increases in GLP-1 had greater food-cue reactivity in the dorsal striatum.

While examining the effects of obesity on the neural processing of food cues has contributed to our understanding of obesity related changes in brain reward pathways, it is important to consider underlying factors, such as dietary intake, that may contribute to increased susceptibility to overeating and obesity. To that end, Burger and Stice recently reported that total energy intake, independent of adiposity, is related to a greater anticipatory response to food within areas in the brain involved in attention and reward processing (62). Our results are in line with these findings and further suggest that habitual consumption of added sugar, accounting for total energy intake, may drive greater striatal reactivity to food cues. Putting these findings in the context of the dynamic vulnerability model of obesity, which suggests that a heightened brain reward response to food cues is associated with greater susceptibility to food cue induced overeating (63–65), these data raise the possibility that high habitual added sugar intake may increase vulnerability to cue related overeating behavior.

Our study design is correlational and does not allow us to determine the directionality of the relationship between added sugar intake and striatal food-cue reactivity (or indeed, whether any causal association exists). Our findings are, however, consistent with experimental animal models that do establish a causal link between excessive sugar intake and greater striatal and behavioral responses to cues for sugar (65). These data could suggest that habitually consuming added sugar affects the brain’s regulation of food reward in a postprandial state, which may lead to overeating. Alternatively, it is also possible that individuals who have greater striatal food-cue reactivity may be motivated to consume more high-sugar foods. Future studies should directly assess this relationship through interventions that experimentally reduce or increase dietary sugar intake in humans. The aim of our study was to examine correlations between habitual added sugar intake, food-cue reactivity in brain reward regions, and endocrine responses by focusing on a fed state induced by a standardized glucose dose. While our approach was to observe such correlations in the glucose and water control conditions separately, a larger sample size may allow hierarchical modeling to directly compare the impact of added sugar intake on hormone and brain responses in the fasted and fed state. Although our data suggest that the relationship between added sugar intake and food-cue reactivity exists only after ingestion of a caloric sugar preload, future analytical approaches directly comparing drink session days would allow testing for interactions, where sample size is not a limitation.

By design, only lean young adults participated in this study since the purpose of this study was to investigate the effects of dietary added sugar in a lean, healthy population. It is possible that obesity status has an additive effect on the relationship between sugar intake, food-cue reactivity, and satiety hormones. Thus, future studies should investigate this relationship in obese individuals, as well as those who are obesity prone due to factors such as genetic predisposition, abnormal eating behaviors, or metabolic dysfunction. We used an ROI-based approach to interrogate the effects of dietary added sugar on responses to food cues in the dorsal striatum and nucleus accumbens based on compelling evidence from animal studies that diets high in sugar and/or fat alter the function of these regions. Future studies should profile the relationship of dietary added sugar intake and food-cue related activity in other relevant regions important for appetite regulation and reward.

This study focused specifically on acute glucose ingestion, but it is notable that “added sugar” typically consists of varying combinations of glucose and fructose, each of which are metabolized differently (66). Recent neuroimaging studies have demonstrated that fructose and glucose differentially affect hormone release, food-cue reactivity in brain reward regions, and resting-state functional connectivity between limbic areas (31, 39, 67, 68). These differences could be investigated in future experiments that seek to define a relationship between acute and habitual sugar intake, perhaps by administration of a disaccharide preload, such as sucrose. In our study, dietary intake data were based on self-reported food intake. The 24-h dietary recall is a widely used method of collecting information about dietary intake, and our data were carefully inspected to exclude over- or underreporting, but we acknowledge that it is an indirect method that may not completely reflect an individual’s dietary habits. Participants in this study completed an average of five 24-h dietary recalls, which allowed us to capture variability in intake and estimate habitual intake of added sugars.

In conclusion, to the best of our knowledge, ours is the first study to show that dietary intake of added sugar is positively correlated with striatal food-cue reactivity, independent of total energy intake, sex, adiposity, or physical activity. Our findings suggest that added sugar intake is related to increased striatal response to food cues, but decreased GLP-1 release following glucose intake. Given that the striatum plays a critical role in mediating reward processing and incentive salience for food cues, these findings suggest that habitual consumption of added sugars may sustain salience of external food cues, even in a postprandial state, which could contribute to susceptibility to overeating and weight gain.

## Ethics Statement

This study was carried out in accordance with the recommendations of the University of Southern California Institutional Review Board. All subjects gave written informed consent in accordance with the Declaration of Helsinki. The protocol #HS-09-00395 was approved by the University of Southern California Institutional Review Board.

## Author Contributions

KP is responsible for conception and design of the work, analysis and interpretation of data, and drafting and editing the manuscript; HD is responsible for acquisition, analysis, and interpretation of data and drafting and editing the manuscript; SL helped with data acquisition, analysis, and interpretation of data and editing the manuscript; JM helped with interpretation of data and editing the manuscript.

## Conflict of Interest Statement

The authors declare that the research was conducted in the absence of any commercial or financial relationships that could be construed as a potential conflict of interest. The reviewers DZ and NS and the handling Editor declared their shared affiliation.
